# A paleoecological context to assess the development of oak forest in Colombia: A comment on Zorilla‐Azcué, S., Gonzalez‐Rodríguez, A., Oyama, K., González, M.A., & Rodríguez‐Correa, H., The DNA history of a lonely oak: *Quercus humboldtii* phylogeography in the Colombian Andes. Ecology and Evolution 2021, doi: 10.100‐2/ece3.7529

**DOI:** 10.1002/ece3.8702

**Published:** 2022-03-23

**Authors:** Henry Hooghiemstra, Antoine M. Cleef, Suzette G. A. Flantua

**Affiliations:** ^1^ Institute for Biodiversity and Ecosystem Dynamics (IBED) University of Amsterdam Amsterdam The Netherlands; ^2^ Bjerknes Centre for Climate Research University of Bergen Bergen Norway; ^3^ Present address: Department of Biological Sciences University of Bergen Bergen Norway

## Abstract

The present “comment” on Zorilla‐Azcué et al.’s paper “The DNA history of a lonely oak: *Quercus humboldtii* phylogeography in the Colombian Andes. Ecology and Evolution 2021, doi:10.100‐2/ece3.7529” provides the paleoecological understanding of oak forest since *Quercus* became apparent in the Northern Andes three glacial–interglacial cycles ago. The interpretation of phylogeographical data is placed in an up‐to‐date paleoecological context. We arrived at sharper conclusions how genetic diversity between *Q*. *humboldtii* populations might have been driven by the dynamic environmental theatre of the recent Pleistocene. This paleoecological context also serves the potential future analyses of other arboreal taxa from the Andean montane forest belt. We show that hypotheses to be tested should grow out of phylogenetic analysis and paleoecological understanding together.

## CONFLICT OF INTEREST

The authors declare no competing interests.

## AUTHOR CONTRIBUTION

**Henry Hooghiemstra:** Conceptualization (lead); Formal analysis (equal); Investigation (equal); Writing – original draft (lead); Writing – review & editing (lead). **Antoine M. Cleef:** Investigation (supporting); Validation (supporting). **Suzette G.A. Flantua:** Formal analysis (equal); Investigation (equal); Validation (equal); Writing – review & editing (equal).

The authors of this study about the lonely oak in Colombia are to be congratulated with the integration of phylogeographical analysis, ecological modeling, and fitting results in a frame of paleoecological understanding of the Northern Andes. Indeed, an integration of results from different disciplines has a high potential to make an incremental progress in understanding current and past environments. In the Northern Andes, paleoecological understanding has made much progress in the last two decades, summarized in Hooghiemstra and Flantua (2019) and visualized in González‐Pastrana et al. (2018). Apart from documentary information in the form of high‐resolution fossil pollen records, showing the anatomy of Pleistocene glacial–interglacial cycles (Bogotá‐A et al., 2015; Groot et al., 2011, 2013), also much progress has been made in understanding mechanisms of speciation in the high Andes (Flantua & Hooghiemstra, 2018; Flantua et al., 2019).

In the Northern Andes, where vegetation change mainly occurred along the altitudinal gradient, the dimensions “altitude” and “time” are as much decisive for distribution patterns as “latitude” and “longitude.” Using the upper forest line (UFL) positions from the last 1 million years as input in a Digital Elevation Model (DEM) of the Northern Andes, the residence time of the UFL in each of the 100‐m intervals has been calculated (Flantua et al., 2019). Most relevant is the new understanding of how rare present‐day warm conditions are, which is as follows: prevailing only during ~15% of the last million years. The most salient consequence is that such understanding should be included in the development and testing of ecological hypotheses which cannot stand on present‐day conditions alone.

Up to 85% of the last million years, current vegetation belts were positioned at lower elevations. While during glacial as well as interglacial conditions, the belt with páramo vegetation showed an altitudinal extension of some 1000 m, the belt with montane forest, including upper montane forest (UMF) and lower montane forest (LMF), was much narrower during glacial times (see fig. 4 in Hooghiemstra & van der Hammen, 2004; Supinfo S1A). At lower elevations in the Northern Andes, land surface is continuous and more abundant than at higher elevations (see fig. 2 in Flantua et al., 2019; Supinfo S1B), allowing the belt of montane forest (UMF and LMF) to be spatially continuous through time (see fig. 10b in Hooghiemstra & Flantua, 2019; Supinfo S1C).

Numerical analyses of the last 1.2 million years (1.2 Ma) of the Funza pollen record from the Bogotá basin (Colombia) showed that the pollen taxonomic composition of forests during different interglacial periods did not vary (see fig. 3 in Felde et al., 2016; Supinfo S1D). This observation provides evidence that a 1500‐m elevational migration of vegetation belts during a full glacial–interglacial cycle implied little risk of losing taxa in the Northern Andes, and likely also in other tropical mountains in general (Flenley, 1979).

**FIGURE 1:**
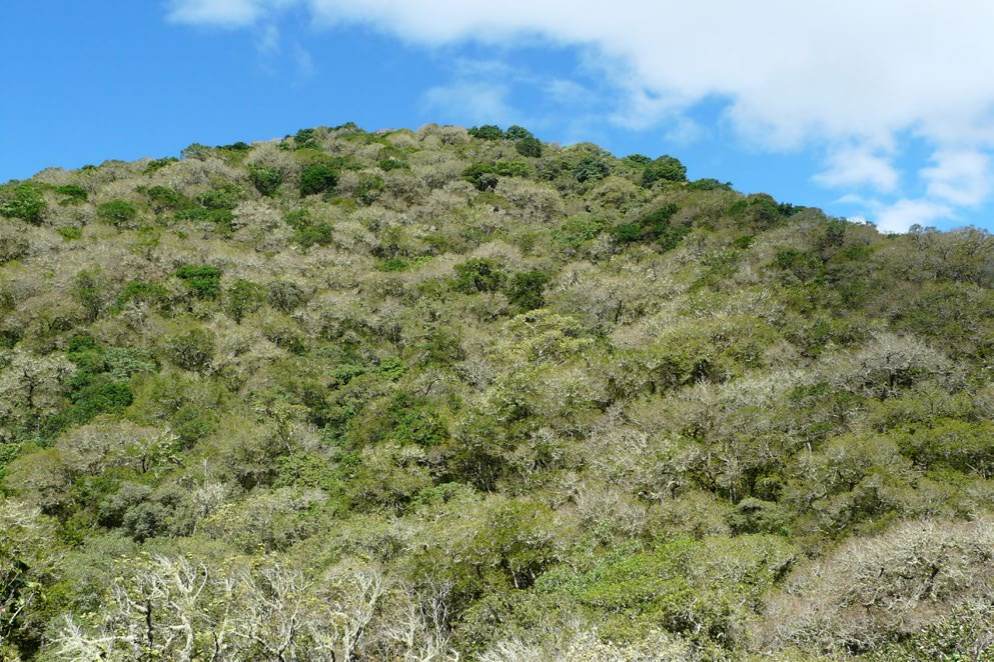
Photograph of dry *Quercus humboldtii* forest at 1600 m elevation in Chachagui, Departemento Nariño, Colombia (photograph courtesy by Andres Avella; photo collection Orlando Rangel, Bogotá)

The ecological range of *Quercus humboldtii* Bonpl. is remarkably large. Along the altitudinal gradient, *Quercus humboldtii* occurs from tropical lowland elevations (>780 m; Avella et al., 2017; Rangel‐Ch & Avella, 2011; Rangel‐Ch, 2017) to ~3250 m (Avella‐M, Dey, et al., 2017; Avella‐M, Rangel‐Ch, et al., 2017; Cleef & Hooghiemstra, 1984; Figure 1). In southern Colombia – at the southernmost limit of the distribution of *Quercus* – oak forests occur up to 3600 m (Becking, 1995). This ca. 2800‐m‐wide elevational interval covers a mean annual temperature (MAT) from ~23 to ~9°C. Along the precipitation gradient, oak forest occurs from dry climatic conditions (mean annual precipitation (MAP) of <1000 mm) to wet conditions (up to 2800 mm MAP; Avella et al., 2017b: 123, 136). Based on floristic composition, two classes in oak forest can be recognized; the *Myrsino coriacea–Querceta humboldtii* forests mainly occurring above 2400 m and the *Billio roseae–Querceta humboldtii* forests found below 2400 m, where MAP values are higher (Avella‐M, Dey, et al., 2017; Avella‐M, Rangel‐Ch, et al., 2017).

This large ecological range must have facilitated *Quercus humboldtii* to pass geographical hurdles along Central America, an area with strongly varying altitudinal profile and intermittent connectivity between populations (O’Dea et al., 2016). It is hypothesized that resistance to warm and dry conditions facilitated *Quercus* to migrate southwards at low elevations through the Panamanian Isthmus, whereas *Quercus’* resistance to a wide temperature range, including the occurrence of night frost, facilitated *Quercus* to also follow migratory pathways at higher elevations (see fig. 9 in Hooghiemstra et al., 2006; Supinfo S1E). Interestingly, in Costa Rica, *Quercus* can be found in both mountainous areas and in the dry Pacific lowlands, while in Panama, all taxa are montane species (Leigh et al., 2014). If we estimate that parts of the Panamanian Isthmus reached elevations of >1000 m altitude by about 3 Ma (O’Dea et al., 2016)—providing a corridor for *Quercus humboldtii* to migrate over ~1000 km to reach the Bogotá area around 0.43 Ma (Torres et al., 2013)—a slow migration process is obvious. During the last 0.43 Ma, *Quercus* extended its distribution some 500 km more southwards, i.e., from the Bogotá area up to the southern border of Colombia (see fig. 2.3 in Hooghiemstra, 2006; Supinfo S1F), likewise exemplifying slow migration. The low migration speed of *Quercus humboldtii* possibly relates to the obligatory relationship with its symbiotic root fungi (Rieske, 2001; Singer, 1963; Wilkinson, 1998). In Colombia, several species of *Quercus* were initially described (Lozano Contreras & Torres Romero, 1965, 1974), but *Quercus humboldtii* Bonpl. remained as the only valid species. The observation that out of the large pool of over 120 *Quercus* species in Mexico (Rzedowski, 1978), only *Quercus humboldtii* Bonpl. succeeded in establishing in South America, may be related to its flexibility to survive a wide range of environments on its way to the southern continent.

Today, *Quercus humboldtii* Bonpl. is mostly observed in the UMF, fueling the idea that oak is most characteristic of the UMF. However, heavy deforestation in the zone of tropical crops (“coffee belt”) causes *Quercus* to be little seen in the zone of the current LMF belt (~1200–2300 m). In addition, the abundance of *Quercus* between 1200 and 2300 m is poorly monitored for the past as this interval with steep slopes has few bogs and lakes, and is poor in pollen records as a consequence (Flantua et al., 2015). However, the past abundance and relevance of *Quercus* in the LMF belt is well illustrated in the pollen record at ~1500 m elevation at Lusitania, located in the province of Cauca on the Pacific slope of the Western Cordillera (Monsalve, 1985). The Holocene interval of this record shows *Quercus* as an important tree in the LMF (see figure 1 in Monsalve, 1985; Supinfo S1G) During last glacial time, when the UFL was below ~2500 m, the Lusitania pollen record shows that forest at ~1400 m was dominated by *Quercus*. Thus, the lower part of the elevational distribution of *Quercus humboldtii* may not have changed since *Quercus* arrived 430.000 years ago (430 ka) in Colombia. The present‐day distribution of *Quercus humboldtii* above 2000 m became established at the start of the Holocene, reflecting a maximal migration over some 1200 vertical meters.

Paleoecological understanding shows a widespread and continuous distribution of montane forest during all ice‐age cycles of the last 2 million years. During the interval from 430 to 250 ka, *Quercus humboldtii* shows a slow but steady increase in its proportion in the montane forest, mainly replacing *Hedyosmum* and *Podocarpus* (see fig. 10 in Torres et al., 2013; Supinfo S1H). During the last three ice‐age cycles, *Quercus* shows a significant presence and therefore the last 250 ka are most relevant. Where other taxa contributing to the montane forest in the Northern Andes are considered, the paleoecology of a much longer time span is relevant for phylogeographical studies. The observations by Zorilla‐Azcué et al. (2021) of “…a high gene flow between populations…” and “…data suggested that connectivity among Andean forest patches was maintained throughout the climatic fluctuations in the late Pleistocene” are in full support of paleoecological understanding. [Here, it is noted that one of the reviewers is of the opinion that any mention about “gene flow” should be prevented as this study by Zorilla‐Azcué et al. (2021) does not include any parameter related to gene flow.] However, the authors’ formulation that “…the persistence of Ceroxylon populations is related to the relatively stable temperature and humidity conditions in Colombia during the last 350 ka…” is misleading. The high‐resolution temperature record from the Northern Andes (Bogotá et al., 2014; Groot et al., 2011, 2013) shows substantial changes in temperature at millennium time scales and larger temperature changes superimposed parallel to changes in climatic humidity. Populations of the palm *Ceroxylon* currently reaching up to 2800 m elevation shifted downslope during colder (glacial) conditions as did all arboreal taxa of the UMF. In this way, *Ceroxylon* continued to occur in “similar” temperature conditions as today (called “climate tracking”).

The authors’ three demographic scenarios shown in figure 3 are most tantalizing to match with current paleoecological understanding as we do not see that the three scenarios proposed reflect likely alternatives.

Scenario 1 assumes a constant effective population size. Contact between populations of *Quercus* occurred at elevations below ~3200 m (during interglacial maxima) to below ~2000 m (during glacial extremes such as the last glacial maximum (LGM)). Interglacial high temperatures prevailed during some 20 kyr during marine isotope stage (MIS) 7, during some 18 kyr in MIS 5e, and during the last ~11 kyr reflecting the Holocene (Groot et al., 2013), all together maximally ~15% of the time. Thus, during ~70% of the time, *Quercus* forest resided in the Colombian Andes at minimally ~800 m while the maximum elevation varied between ~2000 m (extreme glacial) and ~3200 m (extreme interglacial). As a consequence, the total surface of oak forest was most of the time smaller than today and altitudinal fluctuations of the maximum distribution caused repetitive fragmentation and merging of oak populations depending on the mountain profile. The great continuity of surface at lower elevations is predominant but at a local to regional scale fragmentation of oak populations may have occurred during millennia to several tens of thousands of years. From paleoecological understanding, variation in population structure may potentially find its cause in this type of forest dynamics, making scenario 1 very plausible.

Scenario 2 assumes a progressive and constant population expansion. From a paleoecological point of view, this typically occurred at the start of each interglacial during few millennia of climate warming. This scenario does not offer clues to explain the observed variation in population structure.

Scenario 3 assumes a bottleneck event followed by a population expansion and was chosen by Zorilla‐Azcué et al. (2021) as the most plausible scenario. A contraction of the oak population at 47 ka followed by a demographic population expansion at 28 ka was recognized. The authors put forward a climate‐driven explanation (3a), and a floristic composition‐driven explanation (3b). With respect to scenario 3a, the authors observe “…the demographic fluctuations inferred from the molecular data are congruent with equivalent fluctuations in the abundance of Quercus in the fossil record.” Their observation that “…the 47 to 28 ka interval is climatically cool and wet…” is generally correct as shown by the presence of a series of stadials and interstadials reflecting Dansgaard–Oeschger cycles numbered 12 to 7 (see fig. 8 in Groot et al., 2011; Supinfo S1I; fig. 5 in Groot et al., 2013; Supinfo S1J). During the 47–28 ka interval, the UFL shifted between 2500 and 2700 m (see fig. 6 in Groot et al., 2013; Supinfo S1K) and Lake Fúquene (2550 m alt.) was immersed in uppermost ecotone forest without evidence of a marked “contraction” of the population of *Quercus* at this site.

With respect to scenario 3b “biotic interactions” causing a “change in forest composition” constitutes the alternative explanation. Although the authors suggest *Polylepis* to be in competition with *Quercus*, the altitudinal distributions of both taxa hardly overlap (Van der Hammen et al., 2008; see fig. 3 in Groot et al., 2013; Supinfo S1L, and fig. 2 in Wille et al., 2001; Supinfo S1M), making “competition” irrelevant. Pollen records from Lake Fúquene show an expansion of *Polylepis* dwarf forest in the ~45–25 ka interval indeed (see fig. 2.4 in Hooghiemstra, 2006; Supinfo S1N], and fig. 5 in Groot et al., 2013; Supinfo S1J), but this hardly reflects a competition with *Quercus*, rather with uppermost montane forest taxa such as *Weinmannia* and *Miconia*, and with shrubs and herbs of the páramo. Such expansion of *Polylepis* dwarf forest near full glacial conditions is a recurring phenomenon as shown in the long pollen record from the Bogotá basin (Hooghiemstra, 1984; see fig. 4 in Hooghiemstra, 1989; Supinfo S1O; do not mind the incorrect age model in this paper). In summary, a “competition” (explanation 3a) between *Polylepis* (occurring in ecotone forest and as patches in the páramo) and *Quercus* (occurring in the UMF and LMF) is unlikely. There is no evidence of a decline (explanation 3b) in the extension of *Quercus* in the specific period from 47 to 28 ka. However, on local‐to‐regional scales in the Northern Andes, shifts of the UFL of few hundreds of meters may have caused fragmentation of oak populations potentially a driver of the observed genome diversification. In essence, scenario 3b is equivalent to scenario 1, whereas scenarios 3a and 2 do not reflect mechanisms to explain the observed variation and are, therefore, obsolete.

We applaud studies of the distribution of genetic variation within populations and hypotheses being formulated with ecological distribution models, in particular when results are tested against the fossil record. We would like to suggest turning the line of reasoning around and to develop hypotheses that grow out of phylogeographical analyses and paleoecological understanding together. We strongly believe in interdisciplinary research as the present publication shows but we urge future studies to not only make use of papers from other disciplines but also to include corresponding expertise in the research team in order to work with up‐to‐date literature and at the cutting edge of understanding within disciplines.

## Supporting information

Supinfo S1
