# Supplementary material for: A paleoecological context to assess the development of oak forest in Colombia: A comment on Zorilla‐Azcué, S., Gonzalez‐Rodríguez, A., Oyama, K., González, M.A., & Rodríguez‐Correa, H., The DNA history of a lonely oak: Quercus humboldtii phylogeography in the Colombian Andes. Ecology and Evolution 2021, doi: 10.100‐2/ece3.7529
Source: Ecol Evol. 2022 Mar 23;12(3):e8702. doi: 10.1002/ece3.8702 (PMC8941332; doi:10.1002/ece3.8702)
Supplement: Supplementary file 1 — Supinfo S1 [file ECE3-12-e8702-s001.pdf]

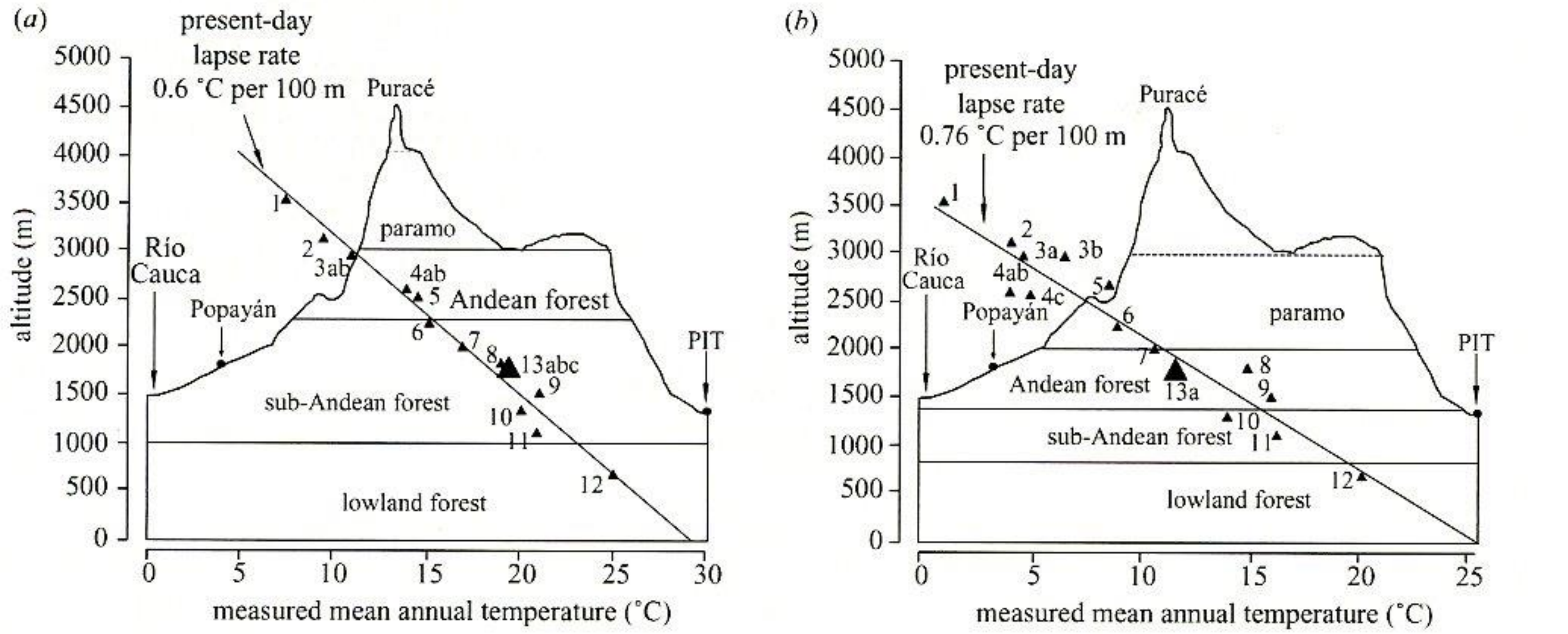

Figure 4. Altitudinal distribution of main vegetation belts along a cross-section in the south Colombian Andes at Popayán latitude. (*a*) Modern vegetation distribution and modern mean annual temperatures at 16 sites. Temperature decrease per 100 m (lapse rate) is *ca.* 0.6 °C. (*b*) Vegetation distribution during the LGM inferred from 18 pollen sites. Temperature decrease per 100 m was *ca.* 0.76 °C which reflects much drier atmospheric conditions. Note: in contrast to the widely used cross-section of Van der Hammen (1974), the lower montane forest belt was maximally compressed during glacial times, suggesting that the lower limit of night frost descended significantly under dry glacial conditions. For an explanation of the sites see Wille *et al.* (2001).

Suppl. Info. A

Fig. 4 in *Hooghiemstra, H., Van der Hammen, T., 2004. Quaternary ice-age dynamics in the Colombian Andes: developing an understanding of our legacy. Phil. Trans. Roy. Soc. Lond. B 359, 173-181.*

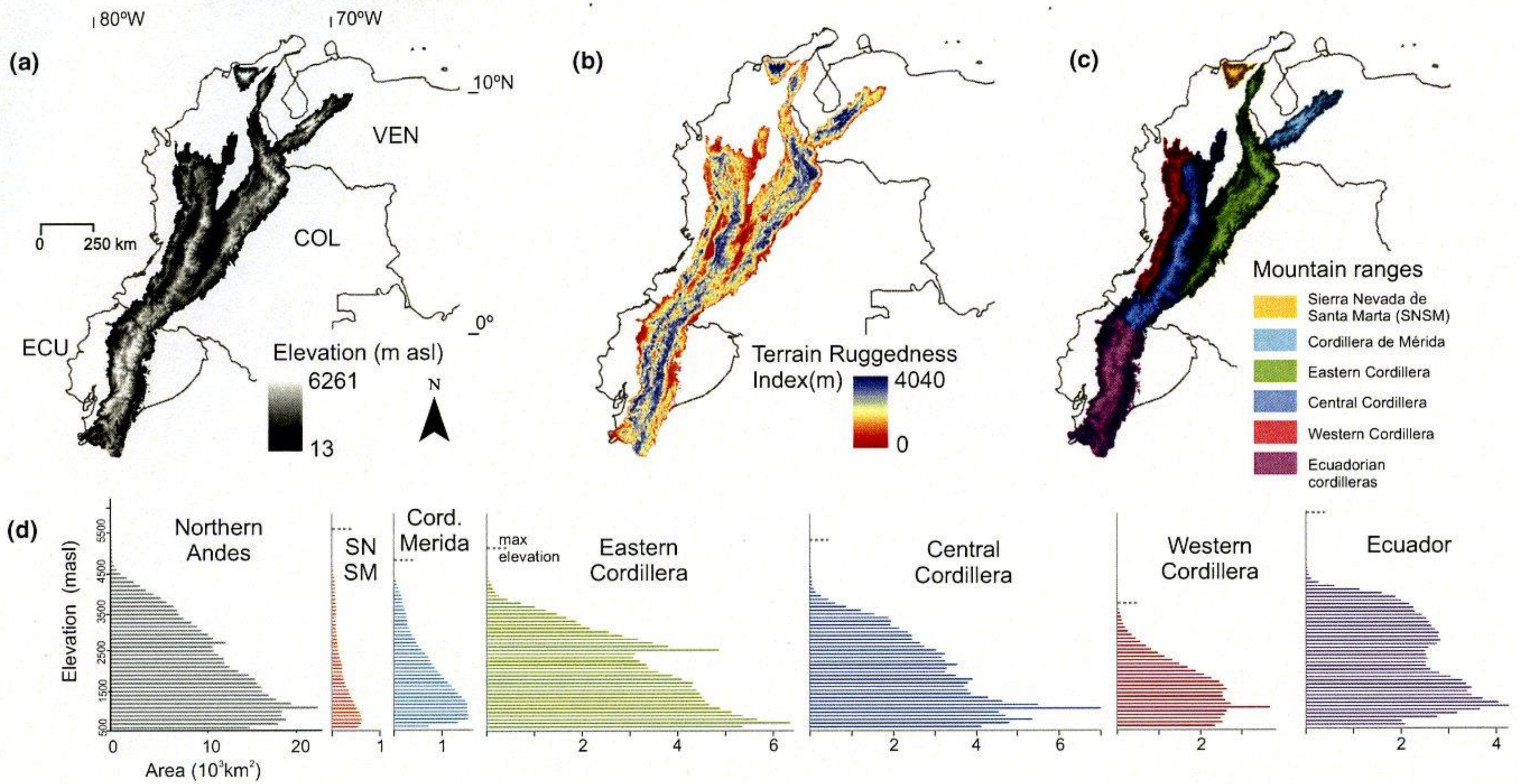

FIGURE 2 Hypsographical curves of the Northern Andes. (a) Elevation (m a.s.l.). (b) Terrain ruggedness index calculates the sum change in elevation between a grid cell and its eight neighbour grid cells (Riley, DeGloria, & Elliot, 1999) using a ca. 30 m DEM (NASA STRM Global 1arc second V003). (c) Delimitation of mountain ranges. (d) Elevational availability of surface area for the Northern Andes and each mountain range separately shown for 100 m bins. Hypsographical curves based on the Shuttle Radar Topography Mission 1-arc second Digital Terrain Elevation Data (~30 m resolution; USGS), taking an elevational threshold of 500 m a.s.l. as the horizontal reference plane. Maximum elevation per cordillera is indicated. VEN: Venezuela; COL: Colombia; ECU: Ecuador

<u>Suppl. Info. B</u>

Fig. 2 in *Flantua, S.G.A., O'Dea, A., Onstein, R., Giraldo, C., Hooghiemstra, H. (2019), The flickering connectivity system of the north Andean páramos J. Biogeogr. 2019, 1-18. doi: 10.1111/jbi.13607.*

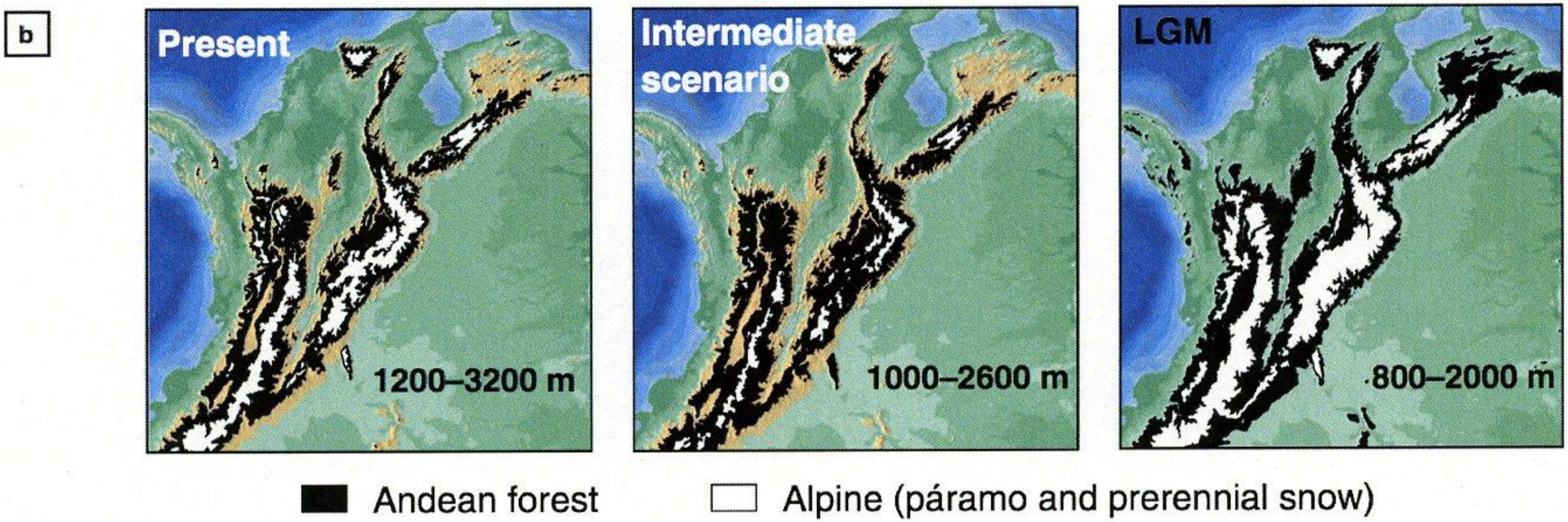

Figure 10. (a) Comparison of the elevational vegetation distribution in the northern Andes for today (left) and during the Last Glacial Maximum at ca. 20 ka (right) showing the maximum change in vegetation cover in a Pleistocene glacial–interglacial cycle. **(b)** Comparison of spatial vegetation distribution of the full montane forest biome, including lower montane forest (sub-Andean forest) and upper montane forest (Andean forest) showing the maximum change in vegetation cover in a Pleistocene glacial–interglacial cycle. Montane forest occurred from ca. 800–2000 m at the Last Glacial Maximum, from ca. 1200–3200 m at present-day, and from ca. 1000–2600 m during an intermediate scenario. (Modified after T. van der Hammen, Journal of Biogeography 1, 1974; Original figure by V. Torres, 2006).

Suppl. Info. C

Fig. 10b in *Hooghiemstra, H., Flantua, S.G.A., 2019. Colombia in Quaternary: an overview of environmental and climatic change (Colombia durante el Cuaternario; una síntesis sobre cambio climático y ambiental). In: Gómez-Tapias, J. (editor), The Geology of Colombia Book, Vol. 4 Quaternary, Chapter 2, pp. 33-84. Servicio Geológico Colombiano https://doi.org/10.2345/tgocb.35.4.2.*

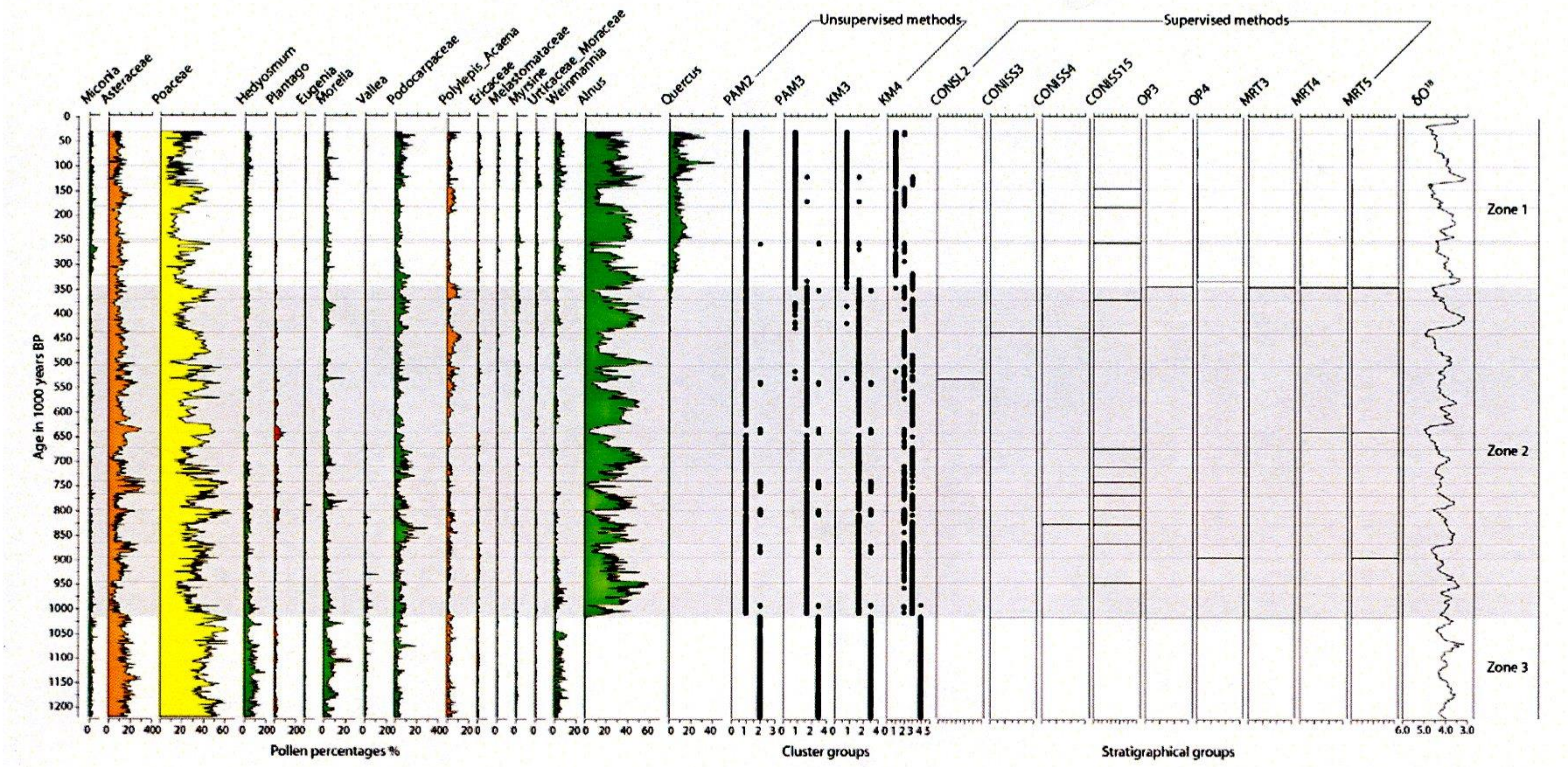

Fig. 3. Summary pollen diagram showing percentage abundances of the selected pollen types *Miconia*, Asteraceae, Poaceae, *Hedyosmum*, *Plantago*, *Eugenia*, *Morella*, *Vallea*, Podocarpaceae, *Polylepis_Acaena*, Ericaceae, *Myrsine*, Urticaceae_Moraceae, *Weinmannia*, *Alnus*, and *Quercus*, the results of the unsupervised partitioning methods (PAM2, PAM3, KM3, and KM4), the results of the supervised clustering and partitioning methods (CONSL2, CONISS3, CONISS4, CONISS15, MRT3, MRT4, MRT5), and the benthic δ O^{18} stack record (Lisiecki and Raymo, 2005). The colours represent different functional groups such as trees (green), shrubs (orange), grass (yellow), and herbs (red). Three major stratigraphical zones are determined based on the consistent results of the supervised partitioning methods using three groups as the appropriate number of clusters and from the results of the CAP analysis below. Dotted lines are included to aid comparison and to distinguish additional zones from the different supervised methods. Abbreviations are: KM = *K*-means partitioning, PAM = Partitioning around medioids, MRT = Multivariate regression tree, OP = Optimal partitioning, CONISS = constrained incremental sum-of-squares, CONSL = constrained single-link cluster analysis, and the numbers behind the abbreviation represent how many groups are being considered. The figure is made using the program C2 version 1.5 (Juggins, 2007a).

<u>Suppl. Info. D</u>

Fig. 3 in *Felde, V.A., Hooghiemstra, H., Torres, V., Birks, H.J.B., 2016. Detecting patterns of change in a long pollen-stratigraphical sequence from Funza, Colombia; a comparison of new and traditional numerical approaches. Rev. Palaeobot. Palynol. 234, 94-109.*

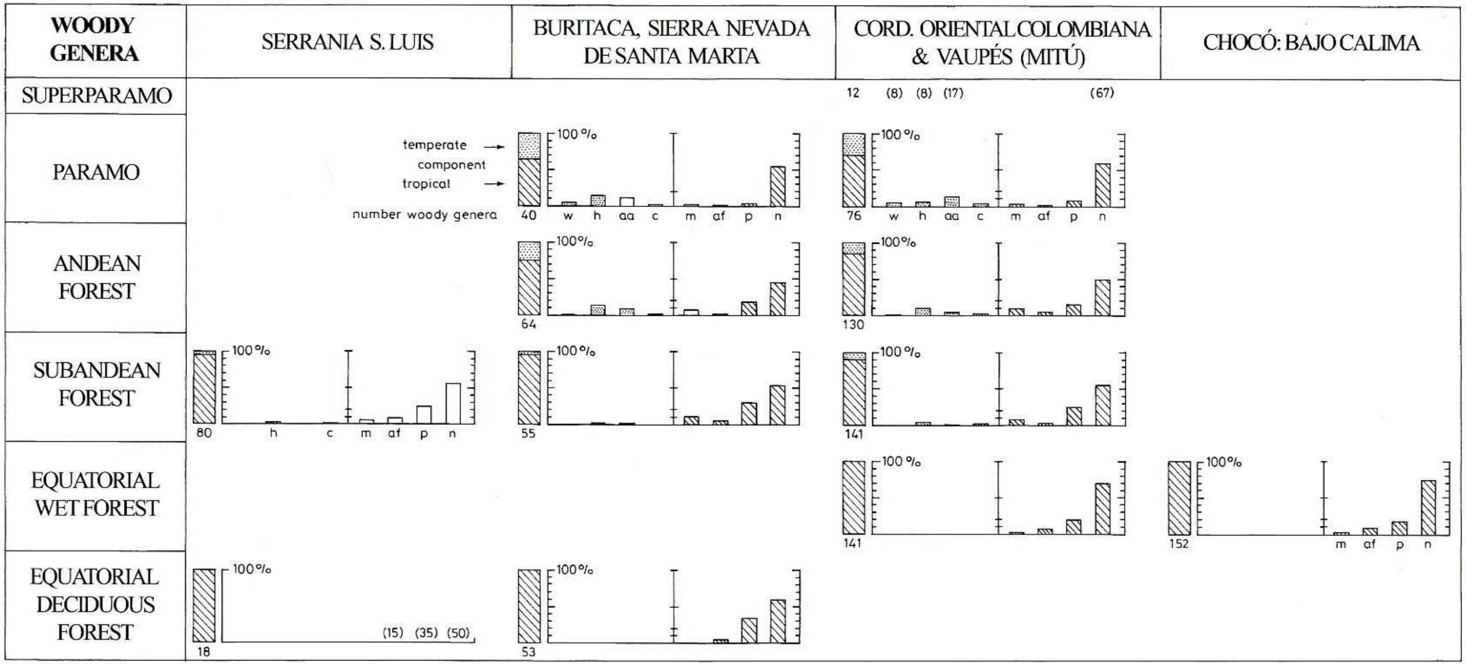

Figure 9. Representation of geographic elements of woody genera for six different vegetation belts in northern Andean and adjacent lowland vegetation showing the adaptation of neotropical lowland taxa to cool climatic conditions. Data are specified (from left to right) for the Serrania San Luis (Venezuela) (subandean forest (1200–1500 m) and deciduous forest (700–1200 m) after Steyermark (1975)); the Buritaca area in the Sierra Nevada de Santa Marta (páramo (> 3300 m), Andean forest (2500–3300 m), and subandean forest (1300–2500 m) based on Rangel & Jaramillo (1984)); Barranquilla (dry deciduous lowland forest after Dugant (1941)); Vaupés (lowland rainforest of Amazonia near Mitú-Caruru, after Cuatrecasas (1958)); the Eastern Cordillera of Colombia (Andean forests after van der Hammen & Jaramillo (unpublished data), and páramos after Cleef (1979) with corrections); and Chocó (Bajo Calima) (lowland rainforest of Amazonia near Trojita-La Brea, after Cuatrecasas (1958)). The bar to the left of each graph shows the number of woody genera and the proportion of tropical and temperate taxa. Phytogeographic elements: n = neotropical; p = pantropical; af = African and American; m = southeast Asian and American; c = cosmopolitan; aa = Austral-Antarctic; h = Holarctic; w = wide temperate. Numbers in parentheses show representation (percentages) of floral elements based on a low total number of genera. After Cleef, unpublished data.

Suppl. Info. E

Fig. 9 in *Hooghiemstra, H., Wijninga, V.M., Cleef, A.M., 2006. The paleobotanical record of Colombia: implications for biogeography and biodiversity. Ann. Missouri Bot. Gard. 93, 297-324.*

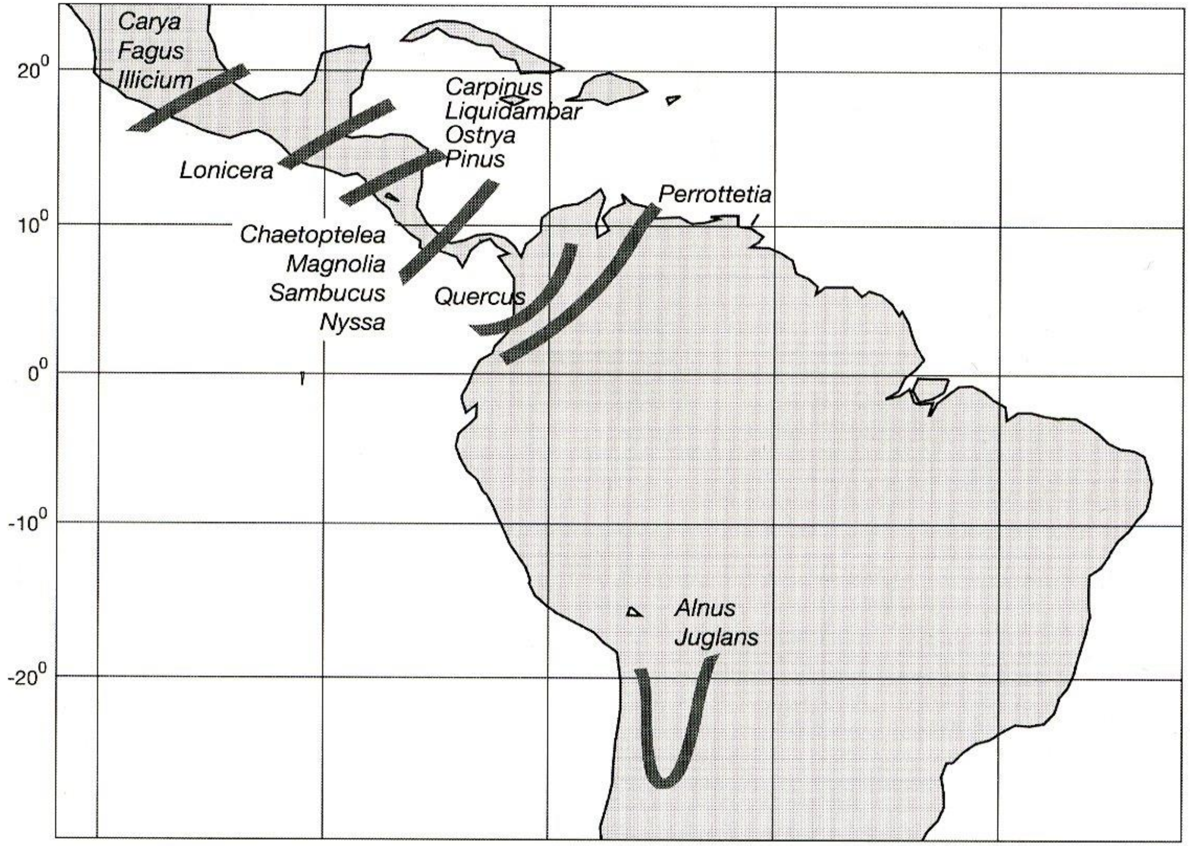

Fig. 2.3. Southward extension of northern hemisphere arboreal taxa in Central America. The southernmost positions of the taxa distribution areas are indicated. Some taxa, such as *Alnus* and *Quercus*, effectively crossed the Panamanian Isthmus and extended their distribution into South America (modified after Webster 1995)

Suppl. Info. F

Fig. 2.3 in ***Hooghiemstra, H., 2006. Immigration of oak into northern South America: a paleo-ecological document. In: Kappelle, M. (editor), Ecology and conservation of neotropical montane oak forests. Studies in Ecology 185, 17-28. Springer, Berlin-Heidelberg, Germany. ISBN 3-540-28908-9.***

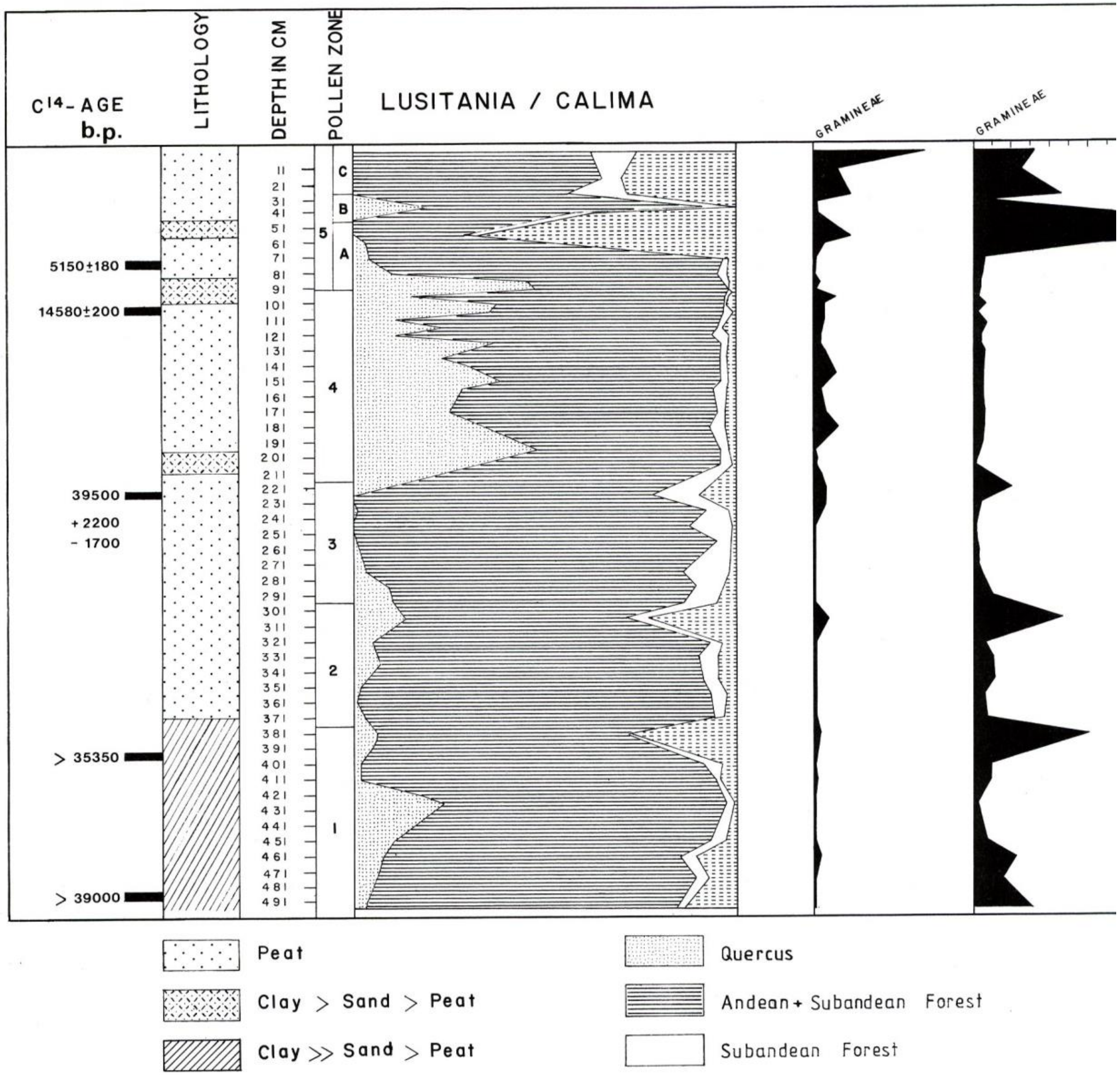

Suppl. Info. G

Fig. 1 in *Monsalve, J.G., 1985. A pollen core from the Hacienda Lusitania. Pro Calima Archaeologisches Projekt in westlichen Kolumbien, Südamerika, Vol. 4, 40-22. Basel. (pdf available)*

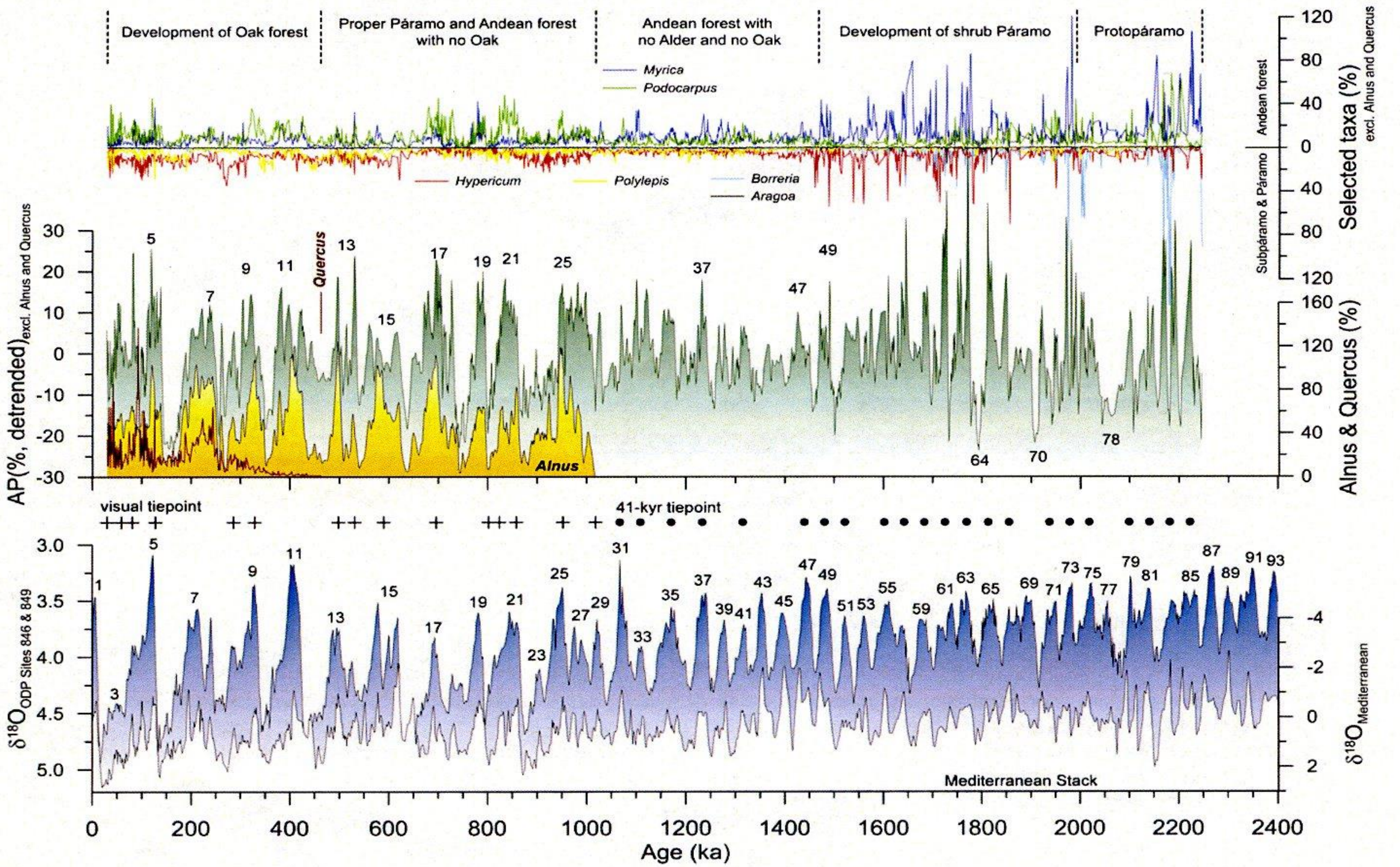

Fig. 10. Pleistocene evolution of montane forest and páramo biomes. Data are plotted following the O-tuning. Bottom panel shows the benthic $\delta^{18}O$ stacked record of ODP Site 846 and 849 (Mix et al., 1995a,b; Shackleton et al., 1995), and the Mediterranean planktic $\delta^{18}O$ stacked record (Lourens et al., 2004). Central panel shows *Quercus*% (dark red line) *Alnus*% record (yellow) and AP% record (detrended; green). Top panel shows the variation in proportions (%) the ecotone tree *Polylepis* (yellow line), the páramo herb *Borreria* (light blue line), the páramo shrubs *Aragoa* (black line) and *Hypericum* (red line), and selected taxa from the Andean forest *Myrica* (blue line) and *Podocarpus* (green line).

<u>Suppl. Info. H</u>

Fig. 10 in *Torres, V., Hooghiemstra, H., Lourens, L.J., Tzedakis, P.C. 2013. Astronomical tuning of long pollen records reveals the dynamic history of montane biomes and lake levels in the tropical high Andes during the Quaternary. Quat. Sci. Rev. 63, 59-72.*

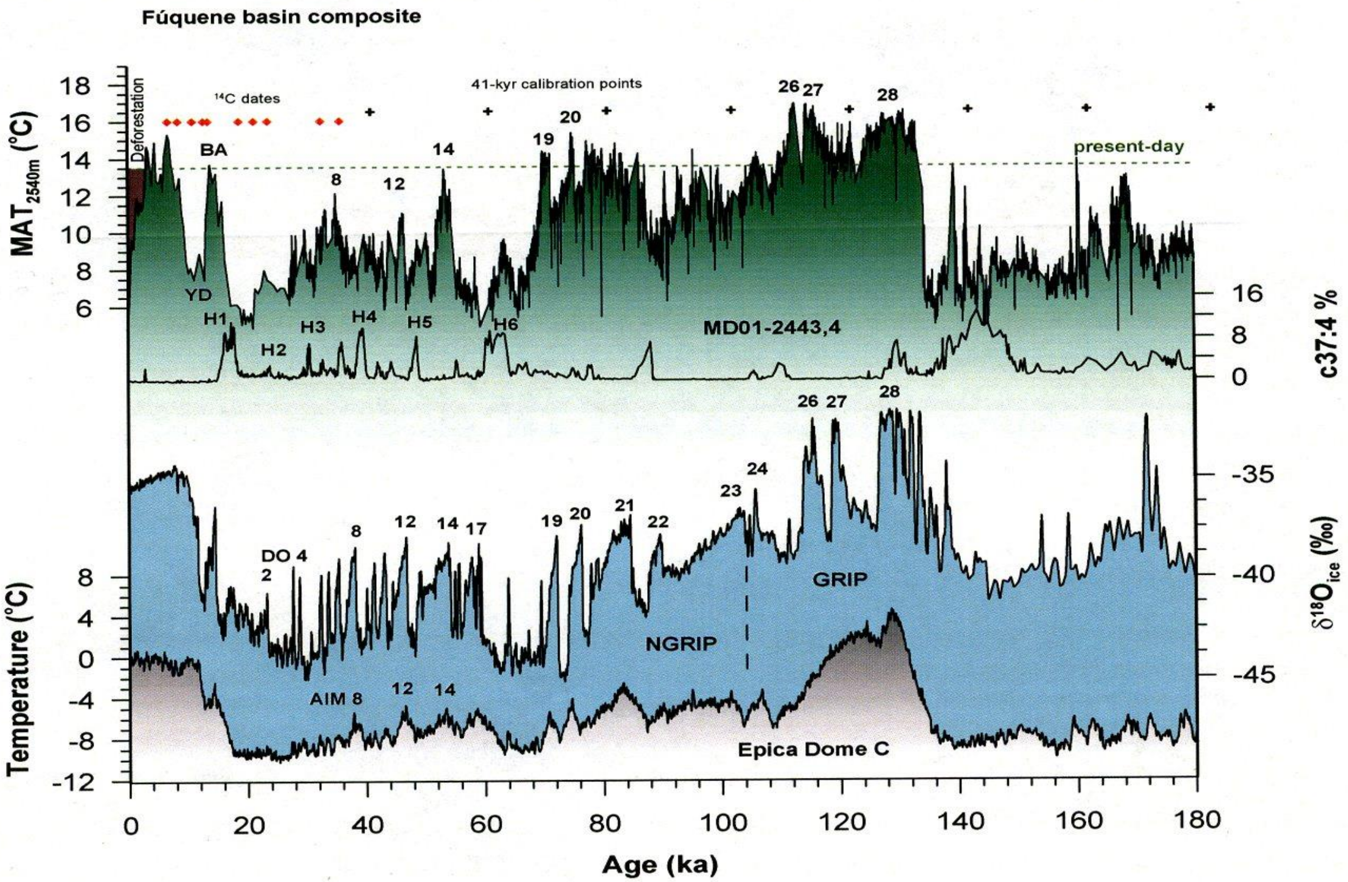

Fig. 8. Comparison between the reconstructed mean annual temperatures (MAT) at Lake Fúquene and the combined Greenland $\delta^{18}O$ ice core records for the past 180 000 years (GRIP-Members, 1993) and temperature record of Epica Dome C (Jouzel et al., 2007; Parrenin et al., 2007). The DO numbers indicate Dansgaard-Oeschger cycles and AIM are the Antarctic Isotope Maxima (AIM). H1-H6 corresponds to the Heinrich events. BA= Bølling-Allerød interstadial and YD = Younger Dryas. The combined Greenland $\delta^{18}O$ record includes (1) the Greenland Ice Core Chronology 2005 (GICC05) (NGRIP Members, 2004) based on annual layer counting for the past 60 ka, (2) the original NGRIP data (NGRIP Members, 2004; Svensson et al., 2008) between 60 and 103 ka, and (3) the data of GRIP below 103 ka.

Suppl. Info. I

Fig. 8 in ***Groot, M.H.M., Bogotá, R.G. Lourens, L.J., Hooghiemstra, H., Vriend, M., Berrio, J.C., Tuenter, E., Van der Plicht, J.H., Van Geel, B., Ziegler, M., Weber, S.L., and 14 Fúquene Project Members, 2011. Ultra-high resolution pollen record from the northern Andes reveals rapid shifts in montane climates within the last two glacial cycles. Climates of the Past 7, 299-316. DOI: 10.5194.cp-7-299-2011.***

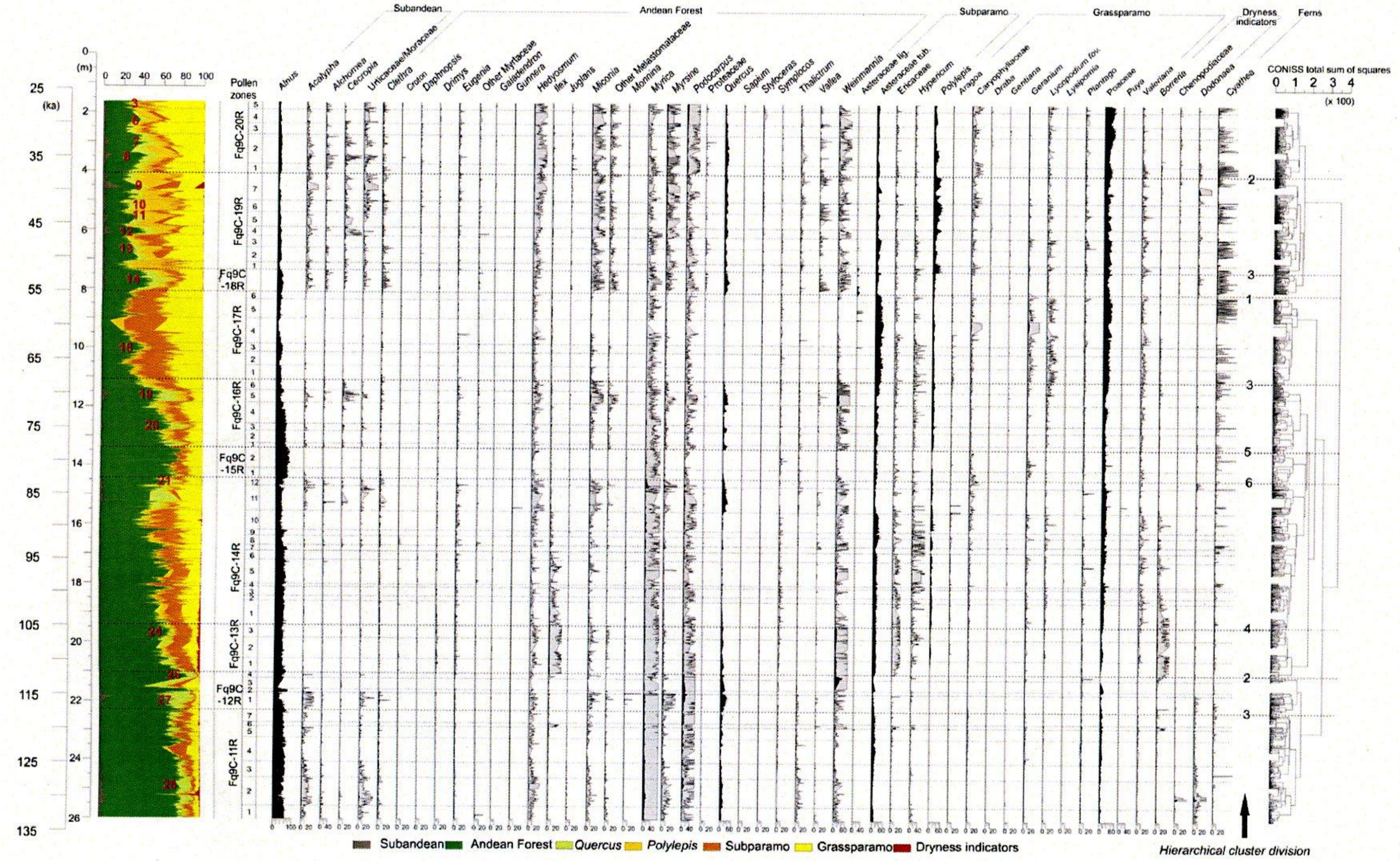

Fig. 5. Pollen diagram of the interval of 1.64–26.00 mcd of core Fúquene-9C showing downcore changes of the regional vegetation. From left to right: estimated age (ka), depth sale (m), main pollen diagram, records of the individual pollen and spore taxa, hierarchical cluster division, and the CONISS cluster dendrogram. Shadows show a 20× exaggeration of curves with a low representation.

Suppl. Info. J

Fig. 5 in *Groot, M.H.M., Hooghiemstra, H., Berrio, J.-C., Giraldo, C., 2013. North Andean environmental and climatic change at orbital to submillennial time-scales: vegetation, water-levels, and sedimentary regimes from Lake Fúquene during 130-27 ka. Rev. Palaeobot. Palynol. 197, 186-204*.

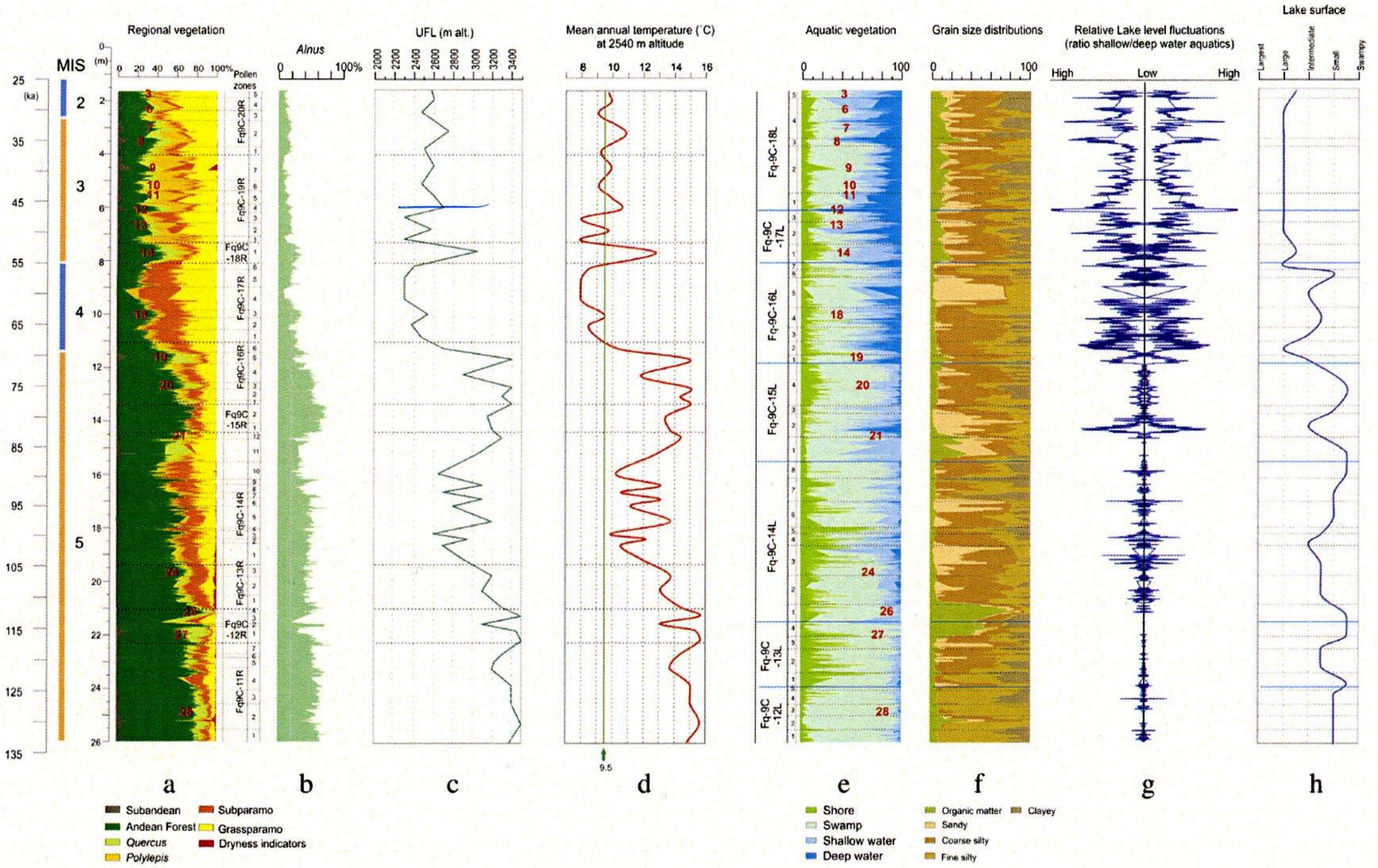

Fig. 6. Summary of environmental change in the northern Andes. See the text for further explanation.

<u>Suppl. Info. K</u>

Fig. 6 in *Groot, M.H.M., Hooghiemstra, H., Berrio, J.-C., Giraldo, C., 2013. North Andean environmental and climatic change at orbital to submillennial time-scales: vegetation, water-levels, and sedimentary regimes from Lake Fúquene during 130-27 ka. Rev. Palaeobot. Palynol. 197, 186-204.*

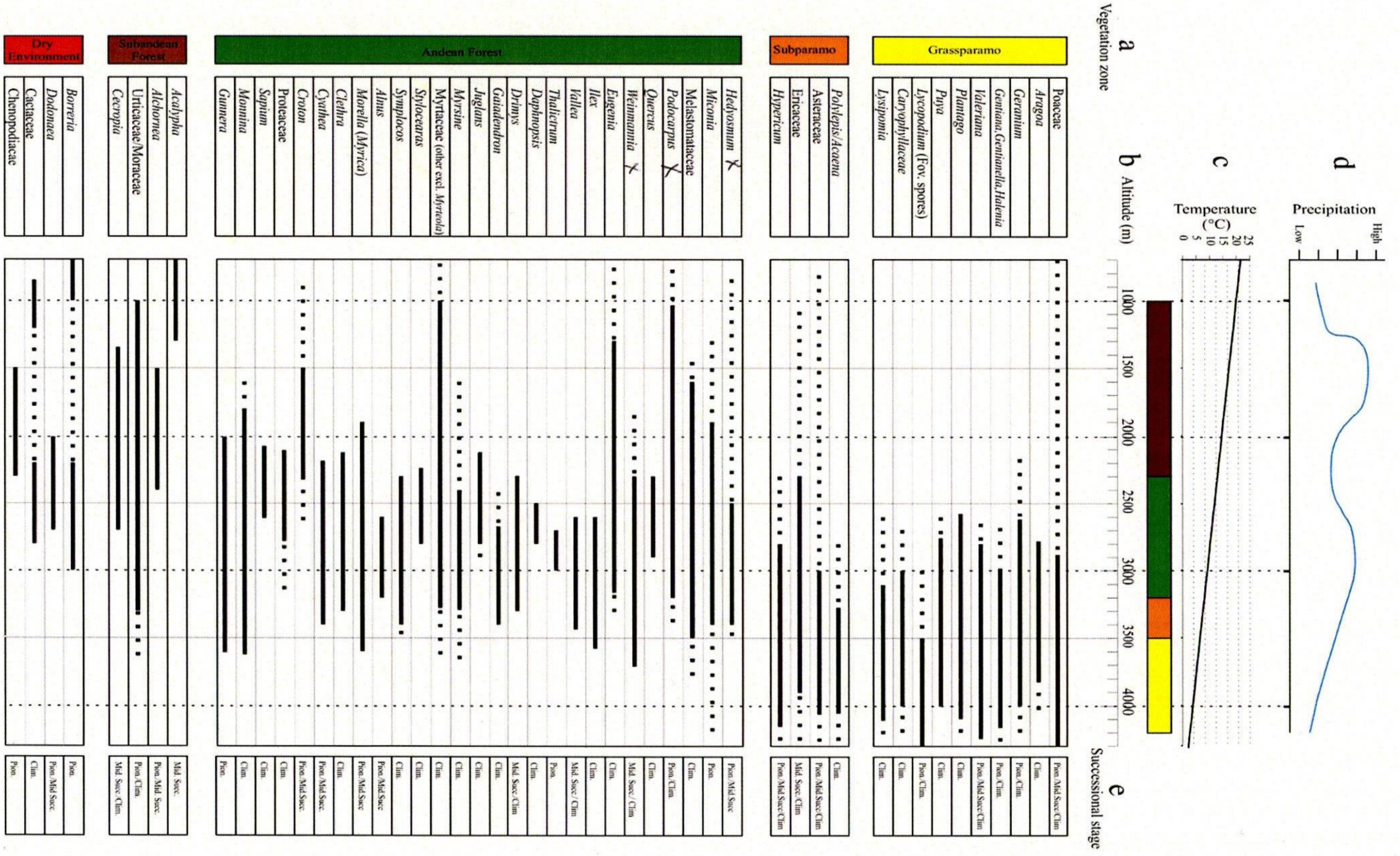

Fig. 3. Altitudinal ranges of selected pollen and spore taxa arranged after ecological preference. (a) Main ecological groups. (b) Modern altitudinal range in the study area; dotted line = full range; solid line = interval of optimum cover. (c) Mean annual temperature (MAT) along the altitudinal gradient. (d) Estimated mean annual precipitation (MAP) along the altitudinal gradient (compiled from the modern vegetation studies mentioned in the text). (e) Main functional place of taxon in the vegetation succession (compiled from the modern vegetation studies mentioned in the text) (Pion: pioneer; Midd. Succ: middle succession; Clim.: climax).

Suppl. Info. L

Fig. 3 in ***Groot, M.H.M., Hooghiemstra, H., Berrio, J.-C., Giraldo, C., 2013. North Andean environmental and climatic change at orbital to submillennial time-scales: vegetation, water-levels, and sedimentary regimes from Lake Fúquene during 130-27 ka. Rev. Palaeobot. Palynol. 197, 186-204.***

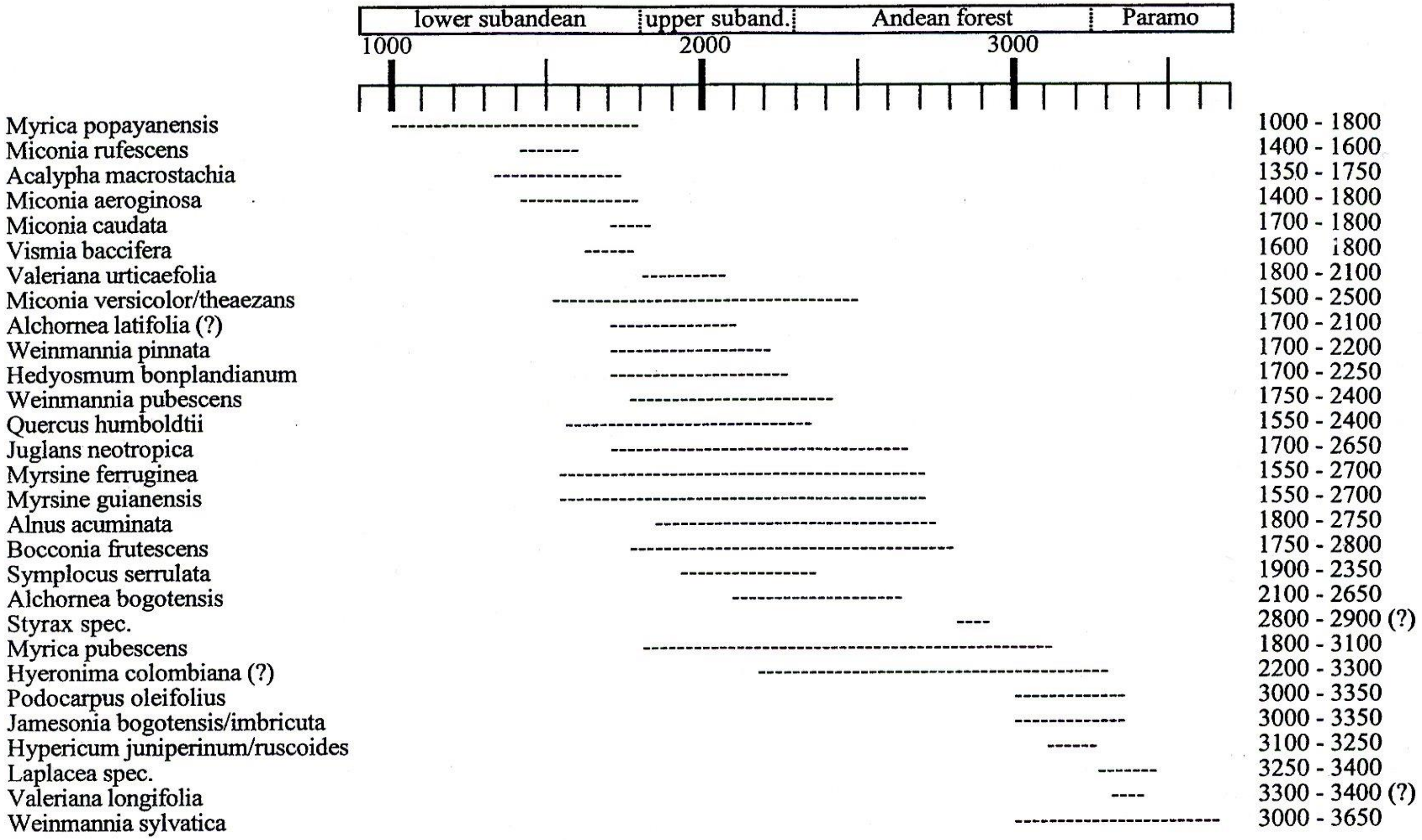

Fig. 2. Altitudinal distribution of modern forest taxa in the Popayán area based on botanical studies by Negret. Only taxa represented in the pollen records are shown (after Wille et al. 2000)

Suppl. Info. M

Fig. 2 in *Wille, M., Hooghiemstra, H., Behling, H., Van der Borg, K., Negret, A.J., 2001. Environmental change in the Colombian subandean forest belt from 8 pollen records: the last 50 kyr. Veget. Hist. Archaeobot. 10, 61-77.*

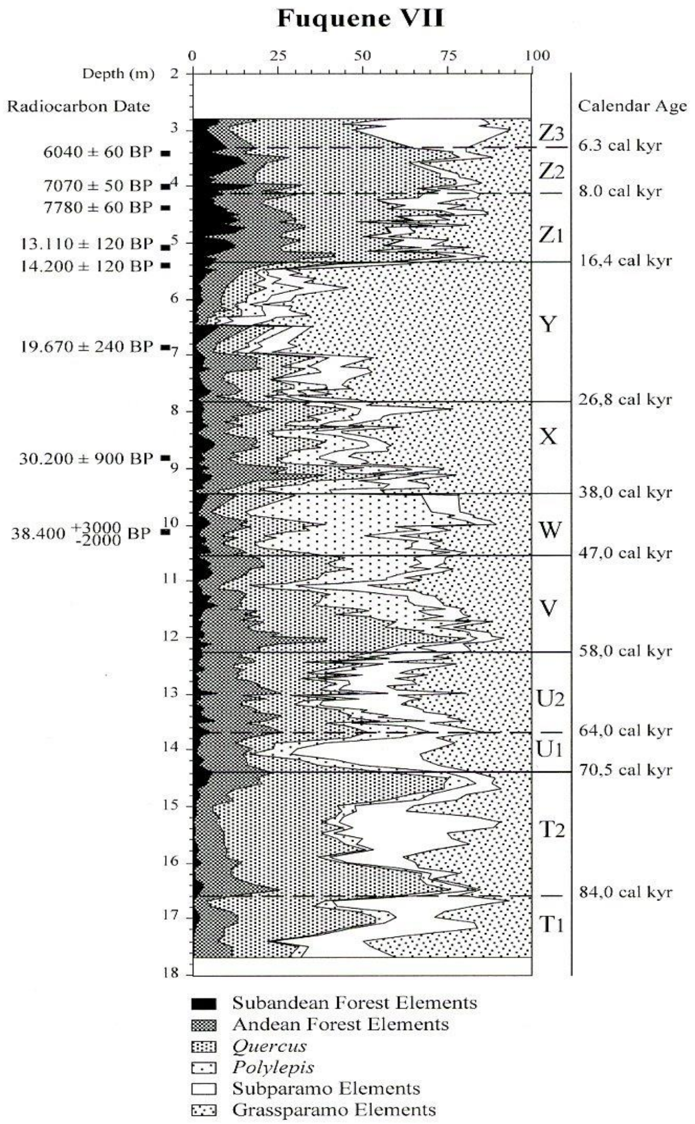

Fig. 2.4. The main pollen diagram of core Fúquene-7C, showing the dynamic vegetation and climate history of the Colombian Andes. Data are given for 5-cm intervals along the core length (about 120-year increments). Laguna de Fúquene is situated at 2,580 m altitude in the present-day Andean forest belt (=upper montane forest belt). The upper forest line (UFL) separates two ecological groups at the right side, and four ecological groups at the left side. The UFL shifted altitudinally from 2,000 m (zone Y) to 3,200 m (zones T2 and Z2). During most of the time period, *Quercus* contributed significantly to the lower and upper montane forests. Temporary replacement by *Polylepis*-dominated forest (zone W) is still insufficiently understood (Mommersteeg and Hooghiemstra 2006)

Suppl. Info. N

Fig. 2.4 in *Hooghiemstra, H., 2006. Immigration of oak into northern South America: a paleo-ecological document. In: Kappelle, M. (editor), Ecology and conservation of neotropical montane oak forests. Studies in Ecology 185, 17-28. Springer, Berlin-Heidelberg, Germany. ISBN 3-540-28908-9.*

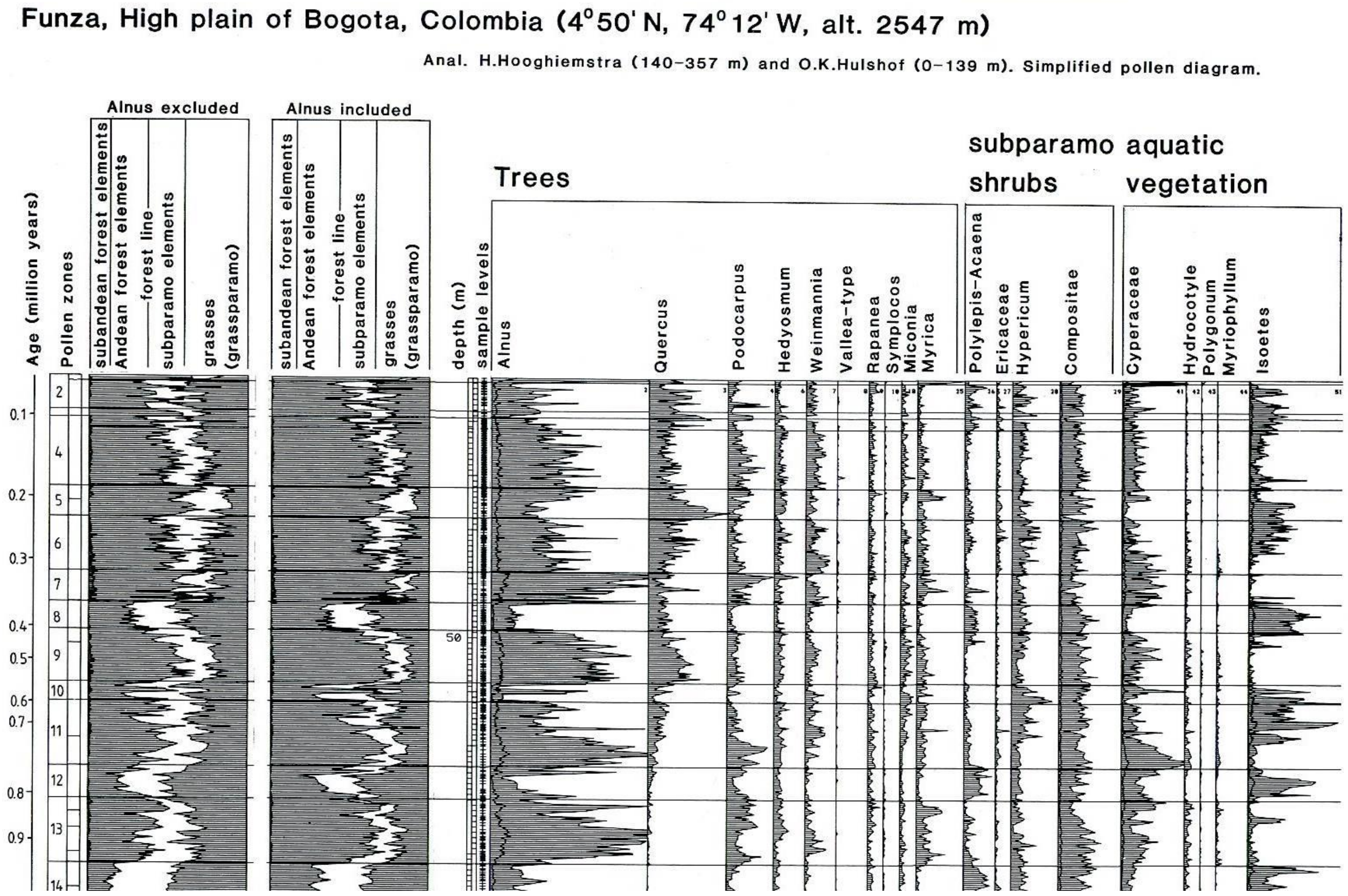

<u>Suppl. Info. O</u>

Fig. 4 in *Hooghiemstra, H., 1989. Quaternary and Upper Pliocene glaciations and forest development in the tropical Andes: evidence from a long high-resolution pollen record from the sedimentary basin of Bogotá, Colombia. Palaeogeogr. Palaeoclimatol. Palaeoecol. 72, 11-26.*
